# Supplementary material for: The Key Role of Tin (Sn) in Microstructure and Mechanical Properties of Ti2SnC (M2AX) Thin Nanocrystalline Films and Powdered Polycrystalline Samples
Source: Nanomaterials (Basel). 2022 Jan 18;12(3):307. doi: 10.3390/nano12030307 (PMC8839355; doi:10.3390/nano12030307)
Supplement: Supplementary file 1 [file nanomaterials-12-00307-s001.zip › nanomaterials-1536179-SI-conversion.pdf]

Supplementary Material

The Key Role of Tin (Sn) in Microstructure and Mechanical Properties of Ti_2SnC (M_2AX) Thin Nanocrystalline Films and Powdered Polycrystalline Samples

Snejana Bakardjieva [1,2,*], Jiří Plocek [1], Bauyrzhan Ismagulov [1,3], Jaroslav Kupčík [1], Jiří Vacík [4], Giovanni Ceccio [4], Vasily Lavrentiev [4], Jiří Němeček [5], Štefan Michna [2] and Robert Klie [6]

[1] Institute of Inorganic Chemistry of the Czech Academy of Sciences, 250 68 Husinec-Rez, Czech Republic; plocek@iic.cas.cz (J.P.); ismagulov@iic.cas.cz (B.I.); kupcik@iic.cas.cz (J.K.)
[2] Faculty of Mechanical Engineering, JE Purkyně University, Pasteurova 1, 400 96 Ústí nad Labem, Czech Republic; stefan.michna@ujep.cz
[3] Department of Inorganic Chemistry, Faculty of Science, Charles University in Prague, Albertov 6, 128 43 Praha, Czech Republic
[4] Nuclear Physics Institute of the Czech Academy of Sciences, 250 68 Husinec-Rez, Czech Republic; vacik@ujf.cas.cz (J.V.); ceccio@ujf.cas.cz (G.C.); lavrentiev@ujf.cas.cz (V.L.)
[5] Faculty of Civil Engineering, Czech Technical University in Prague, Thakurova 7, 166 29 Prague, Czech Republic; jiri.nemecek@fsv.cvut.cz
[6] Department of Physics, The University of Illinois at Chicago, Chicago, IL 60607, USA; rfklie@uic.edu
* Correspondence: snejana@iic.cas.cz

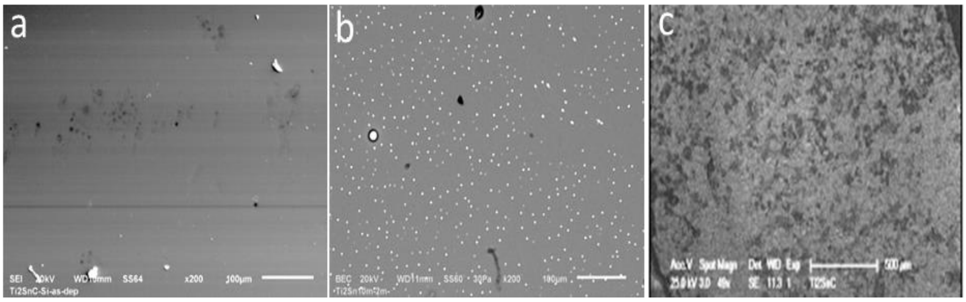

Figure S1. Morphology of Ti_2SnC M_2AX samples (**a**) Ti_2SnC_AGTNCF, (**b**) Ti_2SnC_Ar^+TNCF and (**c**) Ti_2SnC_PPS obtained by SEM with SE detector.

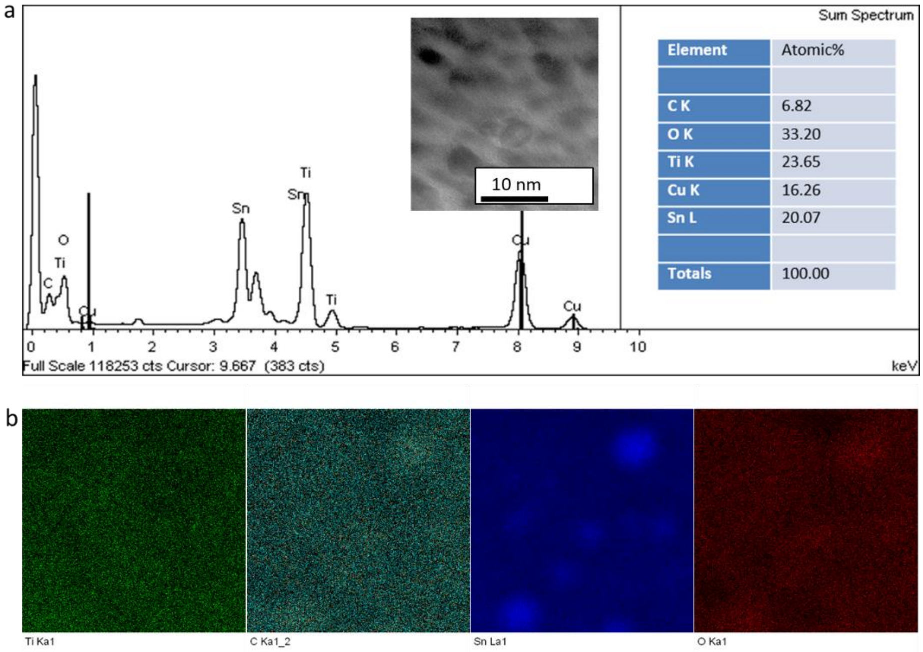

Element	Atomic%
C K	6.82
O K	33.20
Ti K	23.65
Cu K	16.26
Sn L	20.07
Totals	100.00

Figure S2. (**a**) EDS-STEM analysis and (**b**) EDS-STEM mapping Ti_2SnC_AGTNCF. HAADF-STEM image of Ti_2SnC_AGTNCF at low magnification and Table with at. % of elements are present as an inset in Figure S2a.

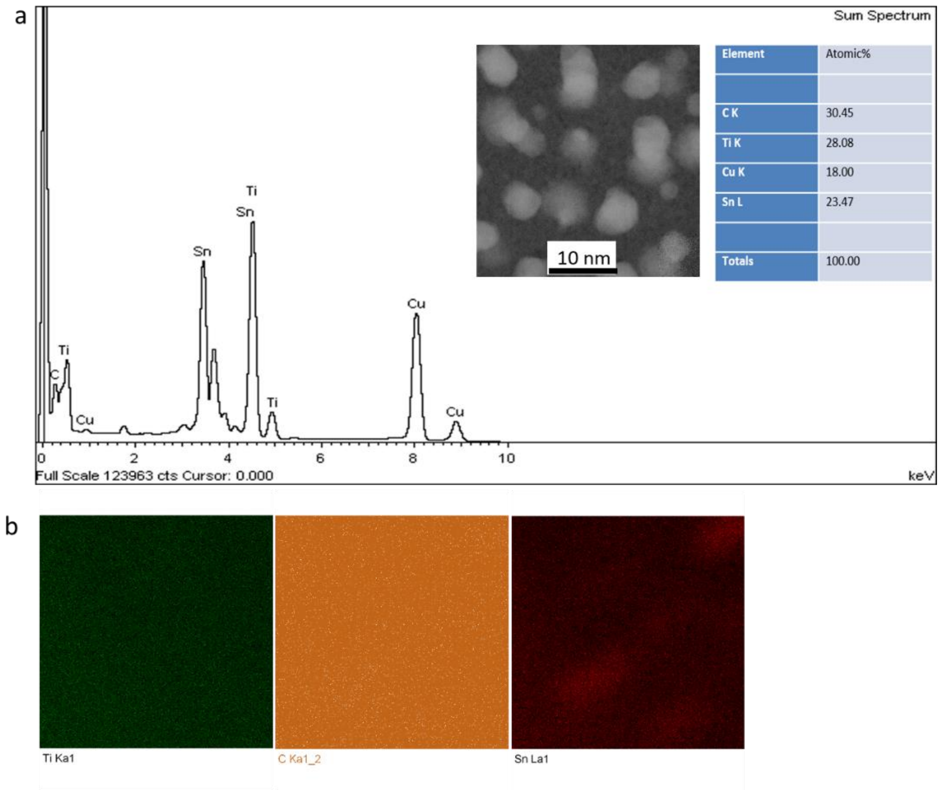

Figure S3. (**a**) EDS-STEM analysis and (**b**) EDS-STEM mapping Ti_2SnC_Ar^{+}TNCF. HAADF-STEM image of Ti_2SnC_Ar^{+}TNCF at low magnification and Table with at. % of elements are present as an inset in Figure S3a.

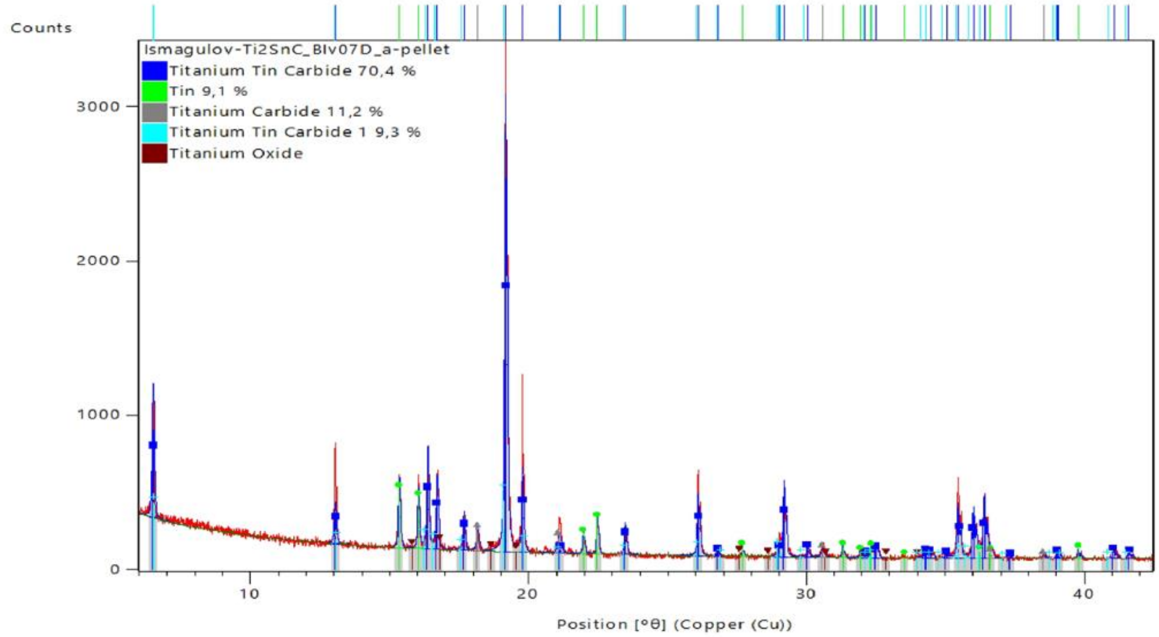

Figure S4. XRD pattern of Ti_2SnC PPS: Titanium Tin Carbide Ti_2SnC – 70.4% Reference code: 04-005-0037, Tin Sn – 9.1% Reference code: 04-004-7745, Titanium Carbide $TiC_{0.55}$ – 11.2% Reference code: 04-018-5143, Titanium Tin Carbide Ti_2SnC – 9.3% Reference code: 04-005-0049.

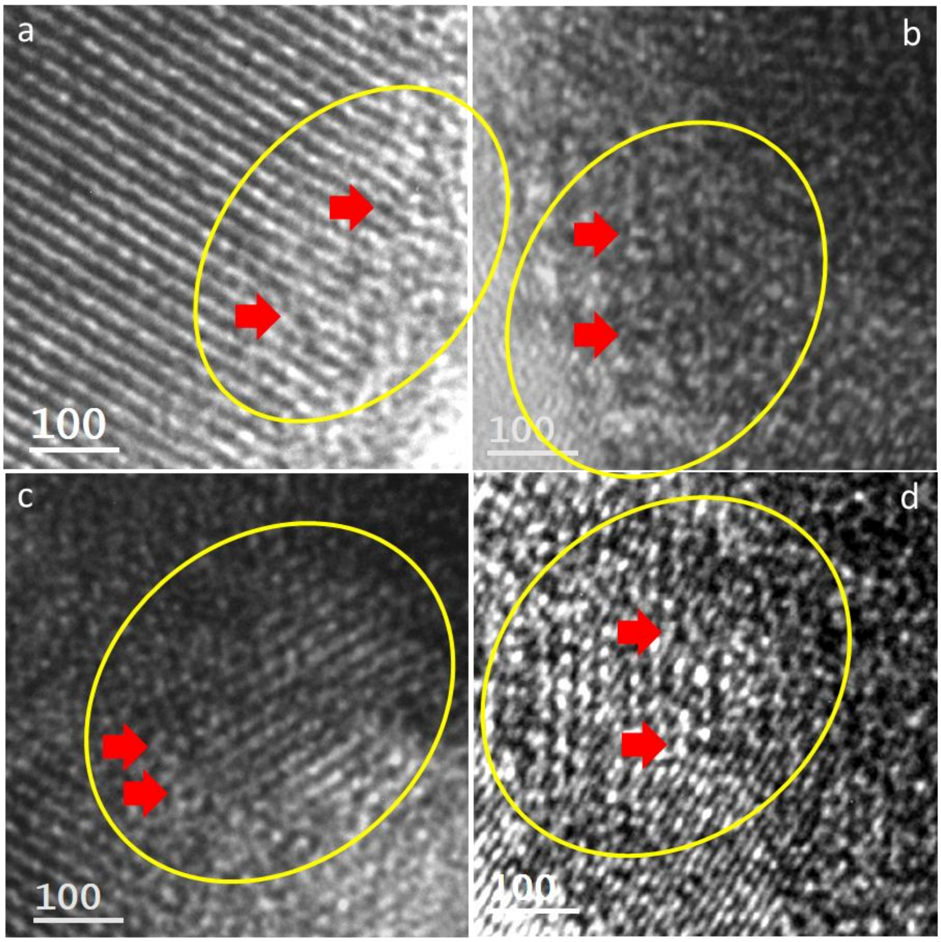

Figure S5. (a–d) HRTEM images of irradiation-induced defect cores in $Ti_2SnC\_Ar^+TNCF$. The yellow marked regions indicated defect cores in a different region of interest, where the interaction of dislocation lines, voids, and atoms segregated on the surface (marked with red arrows) are well visible.

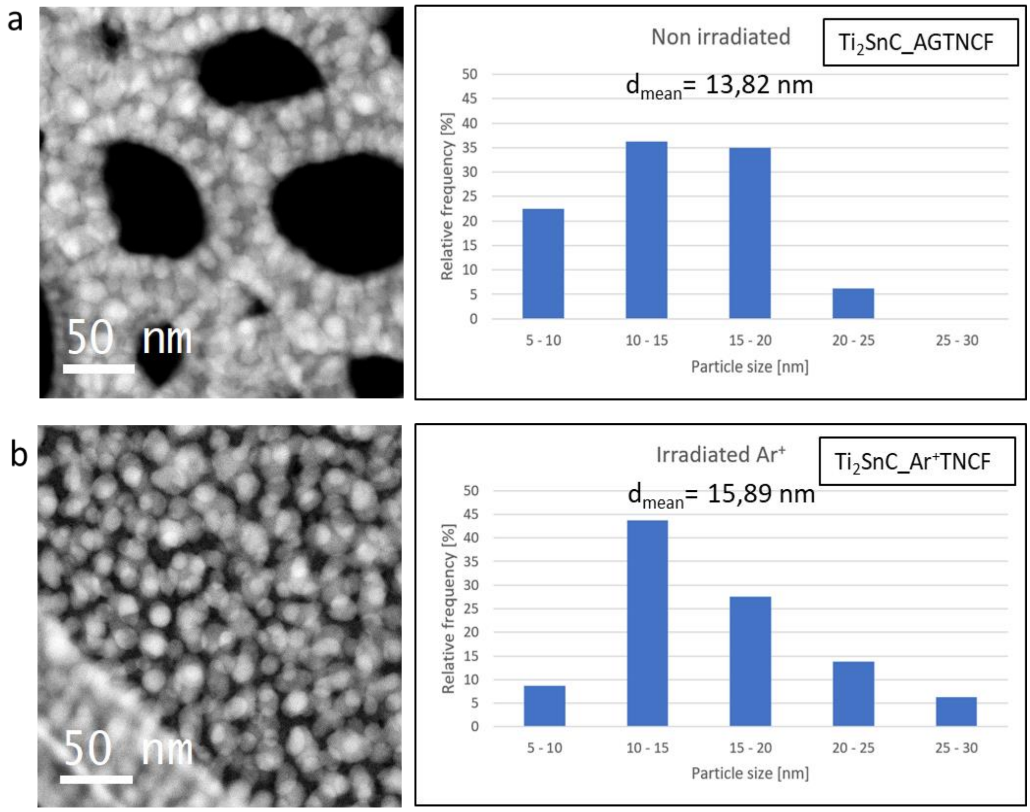

Figure S6. STEM images and corresponding particle size distribution estimated by ImageJ software [1]. (**a**) Ti_2SnC_AGTNCF and (**b**) Ti_2SnC_Ar^+TNCF

References

1. Rueden, C.T.; Schindelin, J.; Hiner, M.C.; DeZonia, B.E.; Walter, A.E.; Arena, E.T.; Eliceiri, K.W. ImageJ2: ImageJ for the next generation of scientific image data. *BMC Bioinformatics*, **2017**, *18*, 529–533. https://doi: 10.1186/s12859-017-1934-z.
